# Progressive interstitial lung disease in patients with systemic sclerosis-associated interstitial lung disease in the EUSTAR database

**DOI:** 10.1136/annrheumdis-2020-217455

**Published:** 2020-09-28

**Authors:** Anna-Maria Hoffmann-Vold, Yannick Allanore, Margarida Alves, Cathrine Brunborg, Paolo Airó, Lidia P Ananieva, László Czirják, Serena Guiducci, Eric Hachulla, Mengtao Li, Carina Mihai, Gabriela Riemekasten, Petros P Sfikakis, Otylia Kowal-Bielecka, Antonella Riccardi, Oliver Distler, EUSTAR collaborators, Marco Matucci Cerinic

**Affiliations:** 1 Department of Rheumatology, Oslo University Hospital, Oslo, Norway; 2 Department of Rheumatology A, Descartes University, APHP, Cochin Hospital, Paris, France; 3 Boehringer Ingelheim International GmbH, Ingelheim am Rhein, Germany; 4 Oslo Centre for Biostatistics and Epidemiology, Research Support Services, Oslo University Hospital - Rikshospitalet, Oslo, Norway; 5 UO Reumatologia e Immunologia Clinica, Spedali Civili di Brescia, Brescia, Italy; 6 VA Nasonova Institute of Rheumatology, Moscow, Russian Federation; 7 Department of Rheumatology and Immunology, Medical School, University of Pécs, Pécs, Hungary; 8 Dipartimento di Medicina Sperimentale e Clinica, Università degli Studi di Firenze, Firenze, Italy; 9 Department of Internal Medicine and Clinical Immunology, Hôpital Claude Huriez, University of Lille, Lille, France; 10 Department of Rheumatology, Peking Union Medical College Hospital (West Campus), Beijing, China; 11 Department of Rheumatology, University Hospital Zurich, Zurich, Switzerland; 12 Department of Rheumatology and Clinical Immunology, University of Lübeck, Lübeck, Germany; 13 Joint Rheumatology Programme, National & Kapodistrian University of Athens Medical School, Athens, Greece; 14 Department of Rheumatology and Internal Medicine, Medical University of Bialystok, Bialystok, Poland; 15 Department of Precision Medicine, Section of Rheumatology, University of Campania Luigi Vanvitelli, Naples, Italy

**Keywords:** scleroderma, systemic, pulmonary fibrosis, autoimmune diseases

## Abstract

**Objectives:**

To identify overall disease course, progression patterns and risk factors predictive for progressive interstitial lung disease (ILD) in patients with systemic sclerosis-associated ILD (SSc-ILD), using data from the European Scleroderma Trials And Research (EUSTAR) database over long-term follow-up.

**Methods:**

Eligible patients with SSc-ILD were registered in the EUSTAR database and had measurements of forced vital capacity (FVC) at baseline and after 12±3 months. Long-term progressive ILD and progression patterns were assessed in patients with multiple FVC measurements. Potential predictors of ILD progression were analysed using multivariable mixed-effect models.

**Results:**

826 patients with SSc-ILD were included. Over 12±3 months, 219 (27%) showed progressive ILD: either moderate (FVC decline 5% to 10%) or significant (FVC decline >10%). A total of 535 (65%) patients had multiple FVC measurements available over mean 5-year follow-up. In each 12-month period, 23% to 27% of SSc-ILD patients showed progressive ILD, but only a minority of patients showed progression in consecutive periods. Most patients with progressive ILD (58%) had a pattern of slow lung function decline, with more periods of stability/improvement than decline, whereas only 8% showed rapid, continuously declining FVC; 178 (33%) experienced no episode of FVC decline. The strongest predictive factors for FVC decline over 5 years were male sex, higher modified Rodnan skin score and reflux/dysphagia symptoms.

**Conclusion:**

SSc-ILD shows a heterogeneous and variable disease course, and thus monitoring all patients closely is important. Novel treatment concepts, with treatment initiation before FVC decline occurs, should aim for prevention of progression to avoid irreversible organ damage.

**Key messages**

**What is already known about this subject?**

- A subset of patients with systemic sclerosis-associated interstitial lung disease (SSc-ILD) develop progressive ILD, which is associated with higher mortality, but the prevalence of progressive ILD and the overall disease course and patterns of SSc-ILD are unknown. Current clinical practice emphasises treatment initiation of SSc-ILD patients with progressive ILD.

**What does this study add?**

- Around 30% of SSc-ILD patients experienced ILD progression during any 12-month period, and 67% of all SSc-ILD patients experienced progression at any time over the mean 5-year follow-up.
- ILD patterns in patients with SSc-ILD are very heterogeneous, with most patients showing both progressive and stable periods.
- Of all progressive SSc-ILD patients, only a minority showed a pattern of rapid, continuously declining forced vital capacity (FVC) with several consecutive episodes of FVC decline and no periods of FVC stability or improvement.

**How might this impact on clinical practice or future developments?**

- These results highlight a pitfall in current clinical practice, where treatment is often initiated after FVC decline has happened, and thus when lung damage has already occurred. Novel treatment concepts are needed and should aim for prevention of progression to avoid irreversible organ damage. This study defines factors that can identify patients at risk for progression. The results also stress the heterogeneity and variability of the course of ILD in SSc, and highlight the need for close monitoring of all patients with SSc-ILD.

## Introduction

Systemic sclerosis (SSc) is a rare autoimmune disease, frequently complicated by interstitial lung disease (ILD), which is associated with worse outcomes.^1–5^ Some patients with SSc-associated ILD (SSc-ILD) develop progressive ILD, showing decline in lung function and/or increasing extent of fibrosis by high-resolution CT (HRCT).^4–10^ The proportion of patients with SSc-ILD who develop progressive ILD and the pattern of disease course in these patients are incompletely understood. Prior analyses of disease course in SSc-ILD have found different disease patterns in different patient cohorts.^8–11^ However, these studies are limited by their small sample size, selected patient populations, significant referral biases and statistical instabilities of the trajectories. Randomised clinical trials provide valuable data, but the 12-month or 24-month duration often used^12–14^ is insufficient for assessment of long-term disease course. There also remains a high unmet need to specifically identify patients with SSc at risk of progressive ILD. Risk factors for SSc-ILD progression have been proposed by several studies;^15–21^ however, their clinical applicability and specific power to predict progression are limited. The optimal combination of risk factors to accurately predict progression has not been identified.

Treatments are available for SSc-ILD, but to date, nintedanib is the only approved treatment shown to reduce lung function decline in patients with SSc-ILD.^14 22 23^ Current clinical practice emphasises treatment of patients with progressive ILD,^24^ and a recent study showed that nintedanib reduces decline of forced vital capacity (FVC) in progressive ILD associated with various underlying conditions, including connective tissue disease-associated ILD.^25^ However, waiting for FVC decline and/or extensive ILD involvement neglects the opportunity of early treatment intervention until after clinically meaningful lung damage has occurred. Novel treatment concepts are therefore aiming to prevent progression and avoid irreversible damage to organs. This requires an understanding of the course and patterns of ILD progression, and reliable prediction algorithms that allow the specific detection of patients at risk of progression at a very early stage. Unfortunately, in SSc-ILD, this knowledge is currently lacking, and treatment initiation is often delayed in clinical practice by waiting for lung function decline over the preceding year before initiation.

The European Scleroderma Trials And Research (EUSTAR) group database is a large, real-world database representative of the general SSc population. It includes a wide range of patients with SSc-ILD, from those with mild and stable to advanced progressive disease.^26 27^

Thus, the aims of this study were: to assess the prevalence of progressive ILD over 12-month periods; to examine disease course and identify patterns of ILD progression in SSc over a 5-year period; and to identify risk factors predictive for progressive ILD in patients with SSc-ILD, using the EUSTAR database.

## Materials and methods

### Study design

Post hoc analyses of prospectively collected patient data from the EUSTAR database were conducted. The structure of the online database, the collected data set and definitions of clinical variables have been described in detail previously.^3 28^

### Patient population and characteristics

Patients registered since 2010 in the EUSTAR database (start of the online version), aged ≥18 years, who fulfilled the 2013 American College of Rheumatology/European League Against Rheumatism SSc classification criteria;^29 30^ with presence of ILD by HRCT or X-ray; recorded disease duration; and with available measurements of FVC and diffusion capacity of the lungs for carbon monoxide (DL_CO_) at baseline and after 12±3 months were included.

### Progressive ILD measured by FVC changes in a 12-month period

To reflect clinical practice with respect to patient follow-up, and the usual study duration in clinical SSc-ILD trials, absolute changes in FVC% predicted were first evaluated over a 1-year period (baseline to 12±3 months).^8 14 31 32^ FVC decline ≥10% predicted is frequently used to define significant ILD progression and was therefore selected in this study as the main outcome measure for progressive ILD. Furthermore, an FVC decline >5% predicted is greater than the estimated minimum clinically important difference at a group level and has previously been associated with increased mortality in SSc.^33 34^ Patients were therefore divided into four progressive ILD subgroups based on absolute change in FVC% predicted from baseline to 12±3 months: significant progression (decline of >10%); moderate progression (decline of 5% to 10%); stable ILD (decline or improvement of <5%); and improvement (improvement of ≥5%) (table 1). The prevalence of annual FVC changes was assessed prospectively in patients with available data over a mean follow-up of 5 years, using the definitions of progressive ILD described above.

**Table 1:** Overall baseline demographic and clinical characteristics of all patients with SSc-ILD and characteristics stratified by ILD progression over the 12±3-month observation period

Progression criteria: ∆FVC% predicted	Total (N=826)	Significant progression (n=100)	Moderate progression (n=123)	Stable (n=396)	Improvement (n=207)
		<−10	−10 to −5	>−5 to <5	≥5
Age, years (SD)*	56 (13.1)	59 (13.1)	56 (12.4)	55 (13.5)	58 (12.4)
Male, n (%)*	150 (18)	17 (17)	16 (13)	81 (20)	36 (17)
Disease characteristics at baseline					
Disease duration, years* (SD)	9.7 (8.3)	8.8 (7.7)	10.2 (8.2)	10.2 (8.5)	8.9 (8.3)
Disease duration <3 years*, n (%)	175 (21)	26 (26)	27 (22)	68 (17)	54 (26)
Diffuse cutaneous SSc, n (%)	365/732 (50)	44/96 (46)	55/106 (52)	182/357 (51)	84/173 (49)
Limited cutaneous SSc, n (%)	367/732 (50)	52/96 (54)	51/106 (48)	175/357 (49)	89/173 (51)
Anti-topoisomerase I Ab, n (%)	421/789 (53)	41/97 (42)	64/117 (55)	218/378 (58)	98/197 (50)
Anti-centromere Ab, n (%)	141/783 (18)	19/97 (20)	18/113 (16)	59/376 (16)	45/197 (23)
Anti-RNA polymerase III Ab, n (%)	23/451 (5)	3/54 (6)	3/60 (5)	10/217 (5)	7/117 (3)
Follow-up period, years*, mean (SD)	5.4 (2.0)	5.8 (1.4)	5.6 (2.0)	4.8 (3.2)	5.0 (3.2)
Lung characteristics					
FVC% predicted,* mean (SD)	87 (21.1)	95 (23.3)	90 (21.8)	85 (20.4)	85 (19.7)
DL_CO_% predicted,* mean (SD)	59 (18.3)	61 (17.8)	60 (17.9)	58 (19.3)	60 (16.8)
∆FVC% predicted,† mean (SD)	–0.1 (10.2)	–18 (7.9)	–7 (1.3)	0.3 (2.2)	12 (7.0)
∆DL_CO_% predicted,† mean (SD)	–0.7 (12.2)	–4 (15.4)	2 (12.8)	–0.3 (10.9)	0.9 (11.9)
NYHA class, n (%)	N=797	n=99	n=119	n=377	n=202
1	363 (44)	44 (44)	57 (46)	167 (42)	95 (46)
2	317 (38)	42 (42)	44 (36)	152 (38)	79 (38)
3	103 (13)	10 (10)	17 (14)	50 (13)	26 (13)
4	14 (2)	3 (3)	1 (1)	8 (2)	2 (1)
Other characteristics					
mRSS, mean (SD)	N=747 10 (8.1)	n=96 11 (7.6)	n=112 10 (8.5)	n=352 10 (7.6)	n=187 10 (8.8)
∆mRSS,† mean (SD)	N=698 –0.4 (4.6)	n=88 0.5 (4.3)	n=103 –0.4 (3.1)	n=337 –0.3 (4.4)	n=170 –1.2 (5.6)
Reflux/dysphagia symptoms, n (%)	547/822 (67)	76/100 (76)	83/122 (68)	261/393 (66)	127/207 (61)
Digital ulcers, n (%)	266/808 (32)	35/100 (35)	38/118 (31)	141/386 (36)	5/2042 (25)
Tendon friction rubs, n (%)	73/804 (9)	7/99 (7)	10/119 (8)	35/383 (9)	21/203 (10)
Synovitis, n (%)	117/810 (14)	18/100 (18)	15/120 (13)	60/386 (16)	24/204 (12)
Muscle weakness, n (%)	182/814 (22)	25/100 (25)	31/120 (25)	78/388 (20)	48/206 (23)
Scleroderma renal crisis, n (%)	11/818 (1)	4/100 (4)	3/120 (2)	6/391 (2)	1/206 (0.5)
ESR, mean (SD)	766 (93) 26 (20.6)	98 (98) 29 (23.9)	115 (93) 25 (21.7)	361 (91) 26 (19.5)	192 (93) 25 (20.2)
Elevated CRP, n (%)	217/797 (27)	40/99 (30)	25/120 (33)	98/377 (26)	49/201 (24)
Immunosuppressant use, n (%)	89/244 (37)	8/20 (40)	8/31 (26)	51/121 (42)	22/72 (31)

Significant progression (FVC decline of >10%); moderate progression (FVC decline of 5% to 10%); stable ILD (FVC decline or improvement of <5%); moderate improvement (FVC improvement of 5% to 10%). Definitions of organ manifestations were described previously.^3 28^ All characteristics were assessed before or on the index date. The following treatment options were received by the included patients at baseline, and for this study were defined as immunosuppressive: prednisone >10 mg/day, azathioprine, cyclophosphamide, mycophenolate, methotrexate or rituximab.

*Available for all 826 patients.

†Change from baseline to 12 months.

Ab, antibody; CRP, C-reactive protein; DL_CO_, diffusion capacity of the lungs for carbon monoxide; ESR, erythrocyte sedimentation rate; FVC, forced vital capacity; mRSS, modified Rodnan skin score; NYHA, New York Heart Association; SSc, systemic sclerosis; SSc-ILD, systemic sclerosis-associated interstitial lung disease.

### Progressive ILD measured by changes in FVC and DL_CO_ over 12 months

A decline in FVC of ≥10%, or a decline in FVC of 5% to 10% along with a decline in DL_CO_ of 15%, is a proposed definition of progressive fibrosis.^8 31 35 36^ Therefore, we also assessed the prevalence of this combined endpoint.

### Disease course and patterns in patients with SSc-ILD, measured by change in FVC from baseline to last available measurement

Because annual FVC patterns can change over time, we evaluated the overall lung function course in patients who had at least two 12-month periods with FVC measurements. These periods could be, but did not need to be, consecutive. For the overall FVC course, patients were divided into five subgroups based on the difference between the first and last available FVC measurement: major decline (FVC decline of >20%); significant decline (FVC decline of >10 and ≤20%); moderate decline (FVC decline of 5% to 10%); stable (FVC decline or improvement of <5%); and improvement (FVC improvement of ≥5%).

The numbers of patients who experienced no 12-month period of decline, one period of decline (moderate or significant) or multiple periods of decline (moderate, significant or both) across all periods with data available over the 5-year follow-up were assessed. Patients with ILD progression were split into different progression patterns according to the number of FVC decline periods: rapid progression (no periods of FVC stability or improvement); progression (more periods of decline than stability/improvement); and slow progression (more periods of stability/improvement than decline).

### Mortality

All-cause mortality was assessed in all patients with SSc-ILD, and in patients with progressive ILD, until last available follow-up.

### Risk factors predictive for progressive ILD

Candidate baseline variables to predict progressive ILD were selected based on reports from the published literature and expert opinion: sex,^15^ age,^16^ reflux/dysphagia symptoms,^17 18^ SSc subtype,^16^ antibody status (anti-topoisomerase antibody (ATA) anti-centromere antibody (ACA), anti-RNA polymerase III antibody (ARA)),^16 19^ baseline FVC,^16 20^ baseline DL_CO_,^16 21^ disease duration,^11 15 37 38^ skin involvement measured by modified Rodnan skin score (mRSS),^16 19 21 39^ erythrocyte sedimentation rate (ESR), C-reactive protein (CRP) level, dyspnoea class, treatment, synovitis and muscle weakness.^16^ Extent of lung fibrosis on HRCT was not included due to extensive missing data.

### Statistical methods

All analyses were performed using IBM SPSS Statistics V.25 and Stata V.15. Pearson χ^2^ test, Fisher’s exact test or independent sample t-test was used, as appropriate. For correlation analyses, Pearson or Kendall’s tau-b coefficients were applied as appropriate. All multivariable analyses were preceded by estimation of correlation between risk factors. Univariable and multivariable logistic regression analyses with OR and 95% CI were applied to analyse the predictive ability of baseline variables for progressive ILD. In the multivariable analyses, 10 events per variable were needed, and the variables were selected by expert opinion.

Univariable and multivariable linear mixed-effect models were performed to identify risk factors of longitudinal changes in FVC (% predicted) over the maximum 5-year follow-up period (baseline, 1, 2, 3, 4 and 5 years). Only patients with at least three serial FVC measurements were included in the analyses. Time and risk factors were fixed effects. Interaction effects between time and fixed factors were checked by including product terms in the models. Only significant interaction terms in the univariable analysis are presented, and they were further included in the multivariable model. Risk factors selected for multivariable analyses were based on expert opinion. All models included random intercept and slope, and an unstructured correlation matrix was used.

### Patient and public involvement

EUSTAR is part of the World Scleroderma Foundation, which has patient representatives from the Federation of European Scleroderma Associations (FESCA) in its governing board.

## Results

### Patient population

Within the EUSTAR database, 6004 patients included since 2010 aged ≥18 years fulfilled the SSc classification criteria and had lung imaging data available. Of these, 2259 (38%) had evidence of SSc-ILD on imaging, of which 826 had valid lung function data available after 12±3 months follow-up and were eligible for inclusion.

Demographic and clinical characteristics of all eligible patients are shown in table 1. No significant difference was observed in the baseline characteristics of the 826 eligible patients and the 1433 ineligible patients (online supplementary table S1).

10.1136/annrheumdis-2020-217455.supp1 Supplementary data

### Prevalence and risk factors of progressive ILD at 12 months

When analysing the prevalence of progressive ILD within the initial 12-month period, we found that 100 patients (12%) had significant ILD progression, 123 (15%) had moderate progression, 396 (48%) were stable and 207 (25%) had improvement.

In multivariable logistic regression analyses, FVC (OR 1.02; 95% CI 1.01 to 1.03; p<0.001), presence of reflux/dysphagia symptoms (OR 1.97; 95% CI 1.14 to 3.40; p=0.016) and mRSS (OR 1.06; 95% CI 1.00 to 1.12; p=0.036) at baseline were predictive for significant ILD progression at 12±3 months. No association was seen with age, sex, disease duration, antibody status, SSc subtype or immunosuppressant treatment.

Prevalence and prediction of progressive ILD measured by the combined FVC and DL_CO_ definition over the initial 12-month period were comparable to these data (online supplementary table S1 and figure S1).

### Disease course and ILD patterns in patients with SSc-ILD

A total of 535 (65%) patients with SSc-ILD had ≥3 FVC measurements available during the mean 5-year (±2.2) follow-up period, allowing for assessment of long-term ILD course. Baseline characteristics did not differ between patients with ≥3 and patients with <3 FVC measurements (n=291 (35%)).

**Figure 1:**
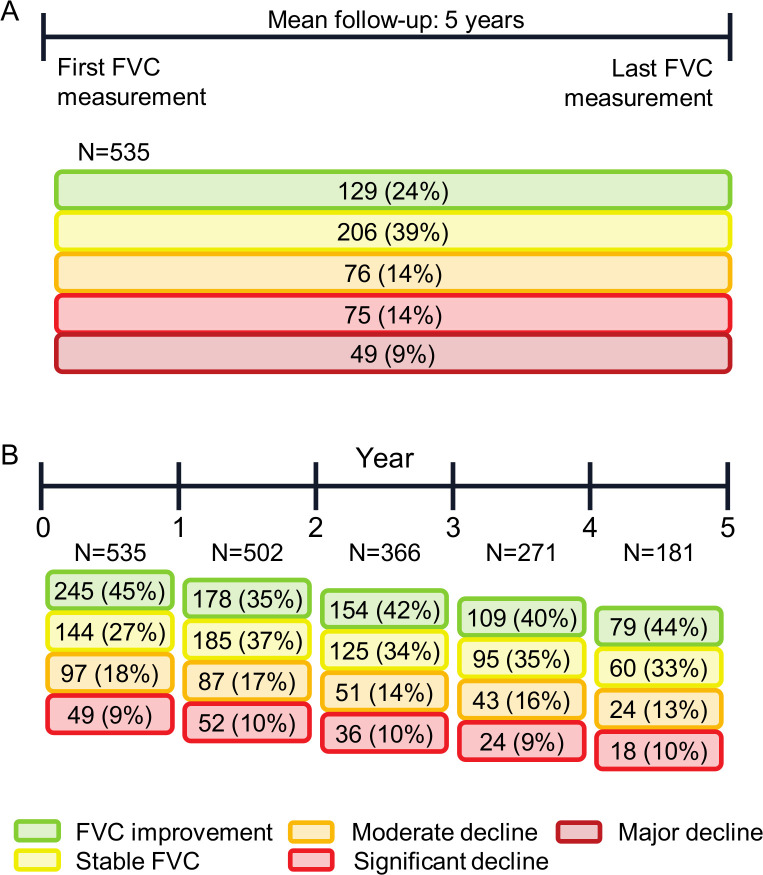
FVC changes among patients with SSc-ILD in the EUSTAR database (number of patients per category): (A) overall change during the 5-year follow-up period; (B) changes during each 12-month follow-up period. (A) Patients for whom ≥3 serial FVC measurements were available were divided into five disease course subgroups based on the overall difference between the first and last FVC measurement (% predicted): major decline (FVC decline of >20%); significant decline (FVC decline of >10% to 20%); moderate decline (FVC decline of 5% to 10%); stable (FVC decline or improvement of <5%); and improvement (FVC improvement of ≥5%). (B) Disease course each year was evaluated by determining the magnitude of FVC changes (% predicted) in each 12-month period during the mean 5-year follow-up defined as follows: significant decline (FVC decline of >10%); moderate decline (FVC decline of 5% to 10%); stable (FVC decline or improvement of <5%); and improvement (FVC improvement of ≥5%). EUSTAR, European Scleroderma Trials And Research; FVC, forced vital capacity; SSc-ILD, systemic sclerosis-associated interstitial lung disease.

**Figure 2:**
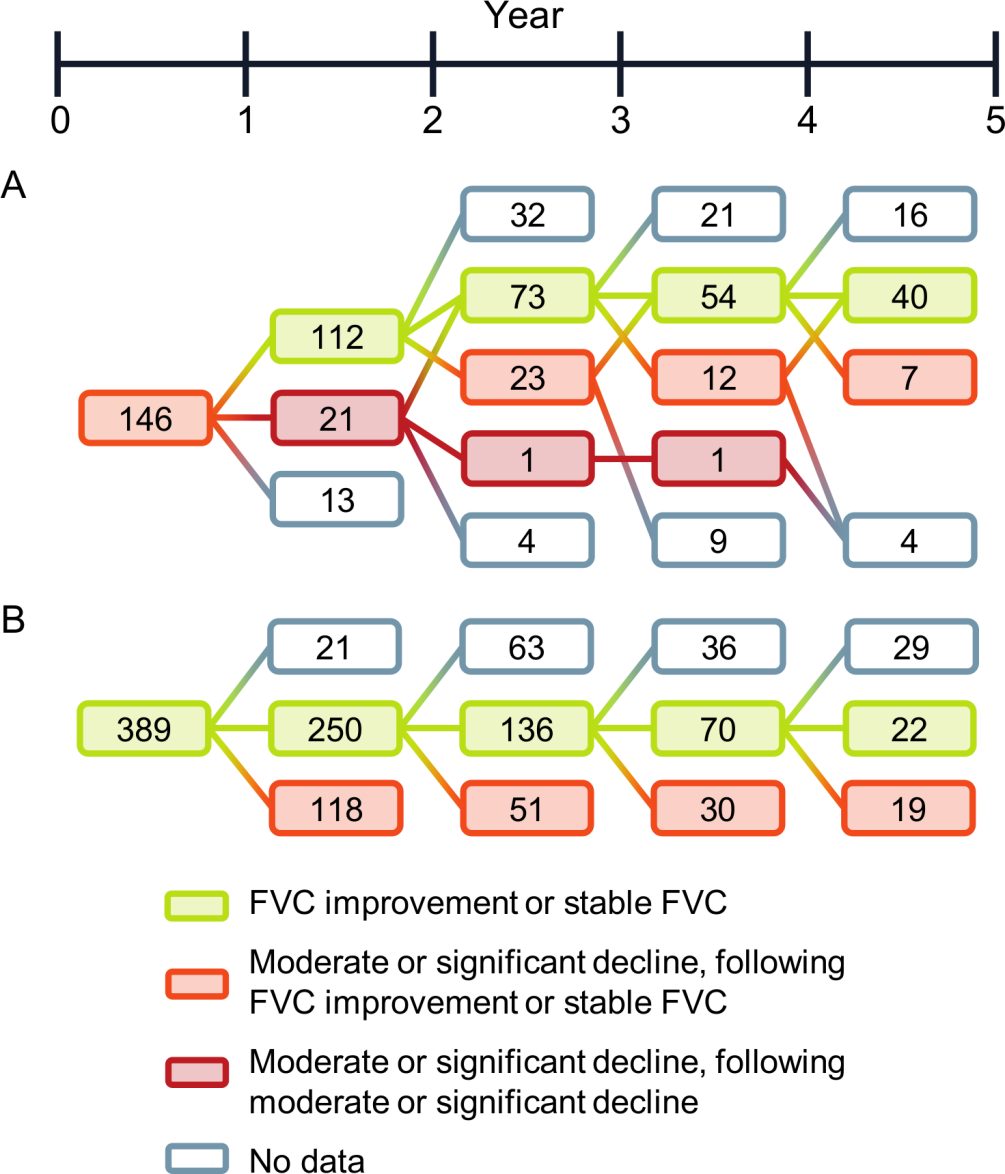
FVC changes in consecutive 12-month periods among patients with SSc-ILD in the EUSTAR database (number of patients per category): (A) subsequent course among patients with stable or improved FVC during the first year of follow-up; (B) subsequent course among patients with minor or moderate decline during the first year of follow-up and those who had further declines. Disease course each year was evaluated by determining the magnitude of FVC changes (% predicted) in individual patients in each 12-month period during the mean 5-year follow-up, defined as follows: significant decline (FVC decline of >10%); moderate decline (FVC decline of 5% to 10%); stable (FVC decline or improvement of <5%); and improvement (FVC improvement of ≥5%). EUSTAR, European Scleroderma Trials And Research; FVC, forced vital capacity; SSc-ILD, systemic sclerosis-associated interstitial lung disease.

To assess the overall disease course, we assessed FVC changes between baseline and last available FVC. We found that 49 (9%) showed major FVC decline (FVC decline of >20%); 75 (14%) had significant decline (FVC decline 10% to 20%); 76 (14%) had moderate decline (FVC decline 5% to 10%); 206 (39%) were stable (FVC changes <5%); and 129 (24%) experienced improvement in FVC (FVC improvement >5%) over the overall disease course (mean 5-year follow-up) (figure 1A). The prevalence of significant ILD progression was between 13% and 18% and the prevalence of moderate progression was between 9% and 10% in each 12-month period over this 5-year follow-up (figure 1B). These progressive periods rarely appeared in consecutive 12-month periods, and progressive periods were mostly followed by stable periods (figure 2A). Stable periods were followed by a progressive period in about 30% of cases (figure 2B). Irrespective of the severity of overall FVC decline (major, significant or moderate), most patients still experienced at least one 12-month period of stable or improving FVC (table 2). On the other hand, patients with stable or improved overall FVC could still experience 12-month periods of FVC decline; these declines were more frequently moderate (FVC decline 5% to 10%) than significant (FVC decline 10% to 20%). Only 178 (33%) patients experienced no period of FVC decline of ≥5% during any 12-month period (table 2).

**Table 2:** Number of patients (n (%)) with SSc-ILD in the EUSTAR database with 12-month periods of FVC decline, stratified by overall FVC decline from first to last available FVC measurement

Overall FVC change from baseline to last FVC		One 12-month period with FVC decline		Two or more 12-month periods with FVC decline		
	No decline (n=178)	Moderate decline (n=113)	Significant decline (n=107)	Only moderate declines (n=65)	One significant and ≥1 moderate decline (n=25)	Only significant declines (n=47)
Improved (n=129)	79 (44)	22 (20)	21 (20)	1 (2)	3 (12)	3 (6)
Stable (n=206)	99 (56)	59 (53)	29 (27)	13 (20)	1 (4)	5 (11)
Moderate decline (n=76)		28 (25)	17 (16)	25 (39)	1 (4)	5 (11)
Significant decline (n=75)		2 (2)	29 (27)	23 (35)	10 (40)	11 (23)
Major decline (n=49)		2 (2)	11 (10)	3 (5)	10 (40)	23 (49)

Overall FVC change from baseline to last FVC: major decline (FVC decline of >20%); significant decline (FVC decline of >10 and ≤20%); moderate decline (FVC decline of 5% to 10%); stable (FVC decline or improvement of <5%); and improvement (FVC improvement of ≥5%).

EUSTAR, European Scleroderma Trials And Research; FVC, forced vital capacity; SSc-ILD, systemic sclerosis-associated interstitial lung disease.

**Figure 3:**
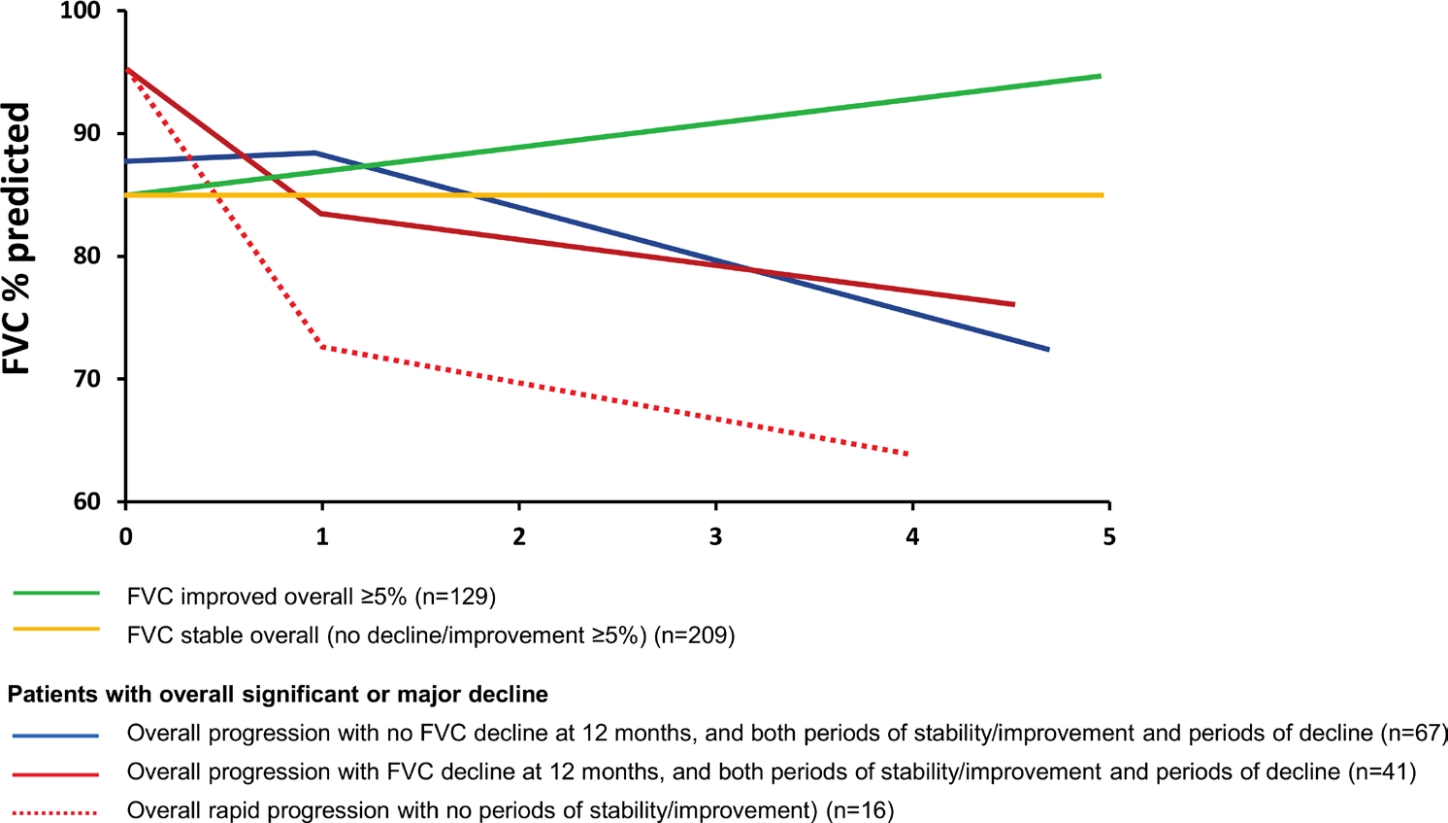
Patterns of disease course in SSc-ILD. Overall disease course was evaluated by determining the magnitude of FVC changes (% predicted) in individual patients from baseline to the end of follow-up defined as follows: major decline (FVC decline of >20%); significant decline (FVC decline of 10% to 20%); moderate decline (FVC decline of 5% to 10%); stable (FVC decline or improvement of <5%); and improvement (FVC improvement of ≥5%). Patterns of disease progression are shown in patients with improved FVC, stable FVC and those with significant or major decline. FVC, forced vital capacity; SSc-ILD, systemic sclerosis-associated interstitial lung disease.

Most patients with SSc-ILD with an overall FVC decline over 5 years had a slow pattern of lung function decline, with more periods of stability/improvement than decline (58%); 34% showed a progressive pattern, with more periods of decline than stability/improvement and slow progression. Only 16 (8%) patients showed a rapidly declining FVC pattern, with several consecutive episodes of FVC decline and no periods of FVC stability or improvement. Patterns of progression in patients with moderate, significant and major overall decline in FVC%, stratified by the presence or absence of a decline in the first 12 months, are shown in figure 3.

### Risk factors predictive of 5-year FVC decline

To identify SSc-ILD patients at risk of ILD progression, we assessed the predictive value of baseline clinical variables on FVC measurements over the 5-year follow-up period. In multivariable linear mixed-effect models, we identified male sex, presence of reflux/dysphagia symptoms and high baseline mRSS as the strongest predictors, with significant interaction effects between time and these variables. This indicates that FVC changed differently over time as a function of one of these predictors (ie, different slopes). Older age, higher DL_CO_, dyspnoea (New York Heart Association class 3 or 4) and increased ESR were also significantly predictive for FVC decline but without a time interaction effect, indicating that the FVC changed significantly over time but not differently between patients with or without these clinical features (table 3). Immunosuppressive treatment was not predictive for FVC decline over time.

**Table 3:** Risk factors for change in FVC over the 5-year follow-up in patients with ≥3 serial FVC measurements in univariable and multivariable linear mixed-effect regression analysis

Predictor variable	Univariable			Multivariable		
	Coefficient	95% CI	P value	Coefficient	95% CI	P value
Time	−0.45	−0.72 to −1.7	0.002	0.8	0.22 to 1.39	0.007
Reflux/dysphagia symptoms	−2.06	−5.06 to 0.94	0.180	0.58	–2.18 to 3.34	0.681
Time×reflux/dysphagia symptoms	−0.76	−1.34 to −0.17	0.011	−0.72	−1.34 to −0.10	0.024
mRSS	−0.51	−0.69 to −0.33	<0.001	–0.31	–0.47 to –0.15	<0.001
Time×mRSS	−0.05	−0.07 to −0.01	0.011	−0.06	−0.10 to −0.02	0.002
Sex	−5.25	−8.91 to −1.59	0.005	–3.90	–7.29 to –0.53	0.024
Time×sex	−0.97	−1.72 to −0.21	0.012	−1.30	−2.10 to −0.49	0.002
Age	0.42	0.31 to 1.53	<0.001	0.47	0.37 to 0.57	<0.001
DL_CO_	0.55	0.47 to 0.62	<0.001	0.45	0.37 to 0.52	<0.001
ESR	−0.14	−0.21 to −0.01	0.001	−0.09	−0.15 to −0.03	0.005
NYHA class	−14.59	−18.7 to −10.49	<0.001	−4.76	−6.59 to −2.92	<0.001
ACA	11.42	7.65 to 15.19	<0.001			
ARA	10.95	1.62 to 20.27	0.021			
ATA	−5.01	−7.98 to −2.05	0.001			
CRP	−7.72	−11.01 to −4.43	<0.001			
dcSSc	−6.37	−7.43 to −3.32	<0.001			

ACA, anti-centromere antibody; ARA, anti-RNA polymerase III antibody; ATA, anti-topoisomerase I antibody; CRP, C-reactive protein; dcSSc, diffuse cutaneous systemic sclerosis; DL_CO_, diffusion capacity of the lungs for carbon monoxide; ESR, erythrocyte sedimentation rate; FVC, forced vital capacity; mRSS, modified Rodnan skin score; NYHA, New York Heart Association.

### Mortality

Of 826 patients with SSc-ILD, 85 (10%) died during follow-up. There were no significant differences in mortality rate between patients with significant ILD progression (11 (12%)), moderate progression (18 (15%)) or stable ILD (36 (9%)) over the initial 12±3-month period. In patients with overall FVC changes measured between baseline and last available FVC, death occurred in 9 of 49 (19%) patients with major decline; 7 of 75 (9%) patients with significant decline; 12 of 76 patients (16%) with moderate decline; 18 of 206 (9%) patients who were stable; and 9 of 129 (7%) patients with improvement, with differences not statistically significant. As there were only a small number of events, no regression analyses were performed.

## Discussion

This is the largest study to prospectively analyse the prevalence of progressive ILD in patients with SSc-ILD, and the first to describe comprehensively the disease course and patterns of ILD progression in patients with SSc over the long term.

The proportion of patients with SSc-ILD who experienced progressive ILD during the initial 12±3-month period was 27%, and in each 12-month period over the mean 5-year follow-up, 23% to 27% of patients experienced progression. These findings are in agreement with estimates of progressive ILD prevalence of 31% to 32% derived from serial lung function data in patients with SSc^6^ and an international physician survey.^40^

Here, we show that patterns of FVC are frequently inconsistent between consecutive 12-month periods. Most patients who experienced an overall decline in FVC had periods of FVC improvement and, conversely, some patients whose FVC improved overall had periods of FVC decline. Patients with overall major FVC decline (FVC decline >20% over the entire study period) usually had several 12-month periods with FVC decline >10% rather than FVC decline of 5% to 10%. Others experienced a slower, but cumulative course of declining FVC. Such patients with slower progression can easily be overlooked in clinical practice and in current treatment strategies that target patients who progress rapidly and with significant FVC changes. Smaller changes in FVC (5% to 10%) may in themselves be clinically significant, as seen in patients with idiopathic pulmonary fibrosis.^41 42^ In clinical practice, this means that FVC decline >5% should alert physicians, especially when multiple declines occur, even when not in consecutive periods. These results highlight a pitfall in current clinical practice, where treatment is often initiated after FVC decline has happened, and thus when lung damage has already occurred.^24^ Novel treatment concepts are needed and should aim for prevention of progression to avoid irreversible organ damage. These results also stress and highlight the need for close monitoring of all patients with SSc-ILD, as also recently suggested by the European expert consensus.^43^ Respiratory symptoms, changes in HRCT findings and desaturation on exercise tests should all be implemented in clinical practice to assess ILD progression and aid treatment decisions.

Robust predictive risk factors are very important for the early identification of progressive patients. Our large, multicentre study demonstrates that skin fibrosis (higher mRSS), male sex and the presence of reflux/dysphagia symptoms are the strongest predictors for FVC decline over time. Other predictive parameters included the presence of inflammation (higher ESR) and shorter disease duration, which are already frequently used as enrichment strategies for clinical studies. These parameters may also be applied in daily clinical practice, helping to identify patients who should receive treatment early, even before the first FVC decline has occurred. However, if earlier treatment of patients at risk for FVC decline truly leads to better outcomes, it needs to be analysed in appropriate randomised controlled clinical trials in the future. Risk factors identified in this study are potential inclusion criteria for such a trial. Interestingly, contrary to our finding that higher FVC at baseline was predictive for ILD progression, previous studies suggested that lower FVC at baseline is a risk factor for progressive ILD.^16 20^ These studies included some SSc patients without ILD, and one study assessed patients within 3 years of SSc diagnosis. Furthermore, definitions of progression (FVC decline of ≥15%,^20^ FVC or DL_CO_ decline of ≥15%, or FVC or DL_CO_ falling below 55%^16^) differed from those in our study. The strongest association with further FVC decline was seen in patients with baseline FVC <65% predicted,^16^ lower than the mean value in our study (86%). Our contrasting findings may reflect these differences and should be assessed in other unselected cohorts.

Strengths of our study include the use of a large set of prospective, representative real-world data, which increases the applicability of our results to clinical practice and different definitions of ILD progression. Nonetheless, this study has several limitations. While the data were gathered prospectively, this was a post hoc analysis. No central lung function reading was conducted, increasing the variability of FVC and DL_CO_. Most patients with SSc-ILD in the database (1433/2259) did not have serial lung function data. Data on immunosuppressant use were only available for 244/826 eligible patients, and the exact date of initiation, treatment indication and cumulative doses are unknown. Several studies^7 8 15^ have suggested that the extent of lung fibrosis by HRCT is prognostic for disease progression and mortality in SSc-ILD. Although data regarding the extent of lung fibrosis were not available in the database for the present analysis, they may be a valuable addition in future studies. Recent analyses also suggest that mRSS progression is an important risk factor for later FVC progression,^39^ which was not analysed in this study. A lead time bias cannot be excluded, as this was not an incident cohort. Finally, ILD-specific mortality was not available in the EUSTAR database. Here, all-cause mortality was not influenced by ILD progression; as recently highlighted,^5^ it is likely that ILD-specific mortality differs between progressive and stable ILD patients.

## Conclusion

This study provides novel insights into the occurrence of progressive ILD in SSc-ILD. The results stress the heterogeneity and variability of the course of ILD in SSc. Close monitoring of patients with SSc-ILD and awareness of the variable course of progression is of high importance in considering when to initiate treatment.
