# Supplementary material for: Progressive interstitial lung disease in patients with systemic sclerosis-associated interstitial lung disease in the EUSTAR database
Source: Ann Rheum Dis. 2020 Sep 28;80(2):219–27. doi: 10.1136/annrheumdis-2020-217455 (PMC7815627; doi:10.1136/annrheumdis-2020-217455)
Supplement: Supplementary data [file annrheumdis-2020-217455supp001.pdf]

Supplementary material

Progressive interstitial lung disease measured by forced vital capacity changes in combination with diffusion capacity of the lungs for carbon monoxide changes over 12-month periods

A decline in forced vital capacity (FVC) of ≥10%, or a decline in FVC of 5–10% along with a decline in diffusion capacity of the lungs for carbon monoxide (DL_{CO}) of 15%, is a proposed definition of progressive fibrosis in patients with interstitial lung disease (ILD), and is predictive of mortality in patients with systemic sclerosis (SSc)-ILD.[8, 31] We therefore also assessed the prevalence of this combined endpoint.

Materials and methods

Patients were divided into five progressive ILD subgroups based on absolute change in FVC and DL_{CO} (% predicted) during the initial 12±3-month period: significant progression (FVC decline of >10%, or FVC decline of 5–10% with DL_{CO} decline of ≥15%); moderate progression (FVC decline of 5–10% with DL_{CO} decline of <15%); stable ILD (FVC decline or improvement of <5%); moderate improvement (FVC improvement of 5–10% with DL_{CO} improvement of <15%); and significant improvement (FVC improvement of >10%, or FVC improvement of 5–10% with DL_{CO} improvement of ≥15%).

Further changes in lung function were evaluated in patients with available data over a mean follow-up of 5 years, using the definitions of progressive ILD described above.

Results

Among the 826 eligible patients with pulmonary function data over the initial 12±3-month period, 113 (14%) had significant ILD progression, 106 (13%) had moderate progression, 408 (49%) were stable, 189 (23%) had moderate improvement, and 10 (1%) had significant improvement (Table S1).

In multivariable logistic regression analyses, higher FVC (odds ratio (OR) 1.03; 95% confidence interval (CI) 1.02–1.04), shorter disease duration (OR 0.97; 95% CI 0.97–0.98), higher erythrocyte sedimentation rate (OR 1.02; 95% CI 1.01–1.02), and presence of reflux/dysphagia symptoms (OR 1.71; 95% CI 1.03–2.84; Figure S1B) were predictive for significant progressive ILD at 12±3 months. Older age, male sex, antibody profile and SSc subtype were not predictive. Further ILD progression over the 5-year follow-up (among patients who had experienced progression during the initial 12±3-month period)

Hoffmann-Vold A-M, *et al. Ann Rheum Dis* 2021; 80:219–227. doi: 10.1136/annrheumdis-2020-217455

Supplemental material BMJ Publishing Group Limited (BMJ) disclaims all liability and responsibility arising from any reliance placed on this supplemental material which has been supplied by the author(s) *Ann Rheum Dis*

was predicted by higher FVC (OR 1.02; 95% CI 1.00–1.04) and shorter disease duration (OR 1.00; 95% CI 1.00–1.02; Figure S1D). The only factor significantly predictive for cumulative progression over 5 years was higher baseline FVC (OR 1.01, 95% CI 1.00–1.02, *p*=0.01).

There were no significant differences in mortality rate between patients with significant ILD progression (14/113 [12%]), moderate progression (15/106 [14%]), or stable ILD (36/408 [9%]) after 12±3 months.

Hoffmann-Vold A-M, *et al. Ann Rheum Dis* 2021; 80:219–227. doi: 10.1136/annrheumdis-2020-217455

SUPPLEMENTARY TABLE S1: Overall baseline demographics and clinical characteristics of all patients with SSc-ILD and those with three serial FVC measurements (who were included in the analyses) or without three serial measurements (who were not included).

	Included (n=826)	Not included (n=1433)	Overall (N=2259)
Age, years (SD)	56 (13.1)	57 (13.4)	57 (13.3)
Male, n (%)	150 (18)	238 (17)	388 (17)
**Disease characteristics**			
Disease duration, years (SD)	9.7 (8.3)	9.9 (8.3)	9.9 (8.3)
Disease duration <3 years, n (%)	175 (21)	294 (21)	469 (21)
Diffuse cutaneous SSc, n (%)	365 (44)	599 (43)	964 (44)
Limited cutaneous SSc, n (%)	367 (44)	668 (48)	1035 (47)
Anti-topoisomerase I Ab, n (%)	421 (51)	700 (52)	1121 (53)
Anti-centromere Ab, n (%)	141 (17)	237 (18)	378 (18)
Anti-RNA polymerase III Ab, n (SD)	23 (3)	34 (5)	57 (5)
Follow-up period, years, mean (SD)	5.4 (2.0)	1.8 (2.2)	2.5 (2.3)
**Lung characteristics**			
FVC% predicted, mean (SD)	87 (21.1)	86 (22.0)	86 (21.7)
DL_{CO}% predicted, mean (SD)	59 (18.3)	59 (20.3)	59 (19.6)
NYHA class, n (%)			
1	363 (44)	527 (38)	890 (41)
2	317 (38)	578 (42)	895 (41)
3	103 (13)	236 (17)	339 (16)
4	14 (2)	31 (2)	45 (2)
**Other characteristics**			
mRSS, mean (SD)	10 (8.1)	10 (8.9)	10 (8.6)
Reflux/dysphagia symptoms, n (%)	547 (66)	905 (64)	1452 (65)
Digital ulcers, n (%)	266 (32)	410 (29)	676 (30)
Tendon friction rubs, n (%)	73 (9)	114 (8)	187 (9)
Synovitis, n (%)	117 (14)	224 (16)	341 (15)
Muscle weakness, n (%)	182 (22)	289 (20)	465 (21)
Scleroderma renal crisis, n (%)	11 (1)	24 (2)	90 (2)
ESR, mean (SD)	26 (20.6)	26 (20.5)	26 (20.5)
Elevated CRP, n (%)	217 (26)	417 (31)	634 (30)
Immunosuppressant use, n (%)	89 (11)	220 (15)	309 (14)

Abbreviations: Ab, antibody; CRP, C-reactive protein; DL_{CO}, diffusion capacity of the lungs for carbon monoxide; ESR, erythrocyte sedimentation rate; FVC, forced vital capacity; mRSS, modified Rodnan skin score; NYHA, New York Heart Association; SD, standard deviation; SSc, systemic sclerosis; SSc-ILD, systemic sclerosis-associated interstitial lung disease.

SUPPLEMENTARY TABLE S2 Overall baseline demographic and clinical characteristics of patients with SSc-ILD and characteristics stratified by ILD progression (defined by FVC and DL_{CO}) over the 12±3-month observation period

	**Total (N=826)**	**Significant progression (n=113)**	**Moderate progression (n=106)**	**Stable (n=408)**	**Moderate improvement (n=189)**
Progression criteria, ∆FVC (∆DL_{CO})		<−10, or −10 to −5 (≤−15)	−10 to −5 (>−15)	>−5 to <5	5 to 10 (<15)
Age, years (SD)	56 (13.1)	59 (13.4)	55 (12.1)	55 (13.5)	58 (12.1)
Male, n (%)	150 (18)	18 (16)	17 (16)	81 (20)	34 (18.0)
**Disease characteristics at baseline**					
Disease duration, years (SD)	9.7 (8.3)	9.2 (7.6)	9.6 (8.4)	10.2 (8.3)	9.0 (8.6)
Disease duration <3 years, n (%)	175 (21)	27 (24)	27 (26)	70 (17)	50 (26.5)
Diffuse cutaneous SSc, n (%)	365 (44)	51 (45)	45 (43)	186 (46)	78 (41.3)
Limited cutaneous SSc, n (%)	367 (44)	58 (51)	43 (41)	180 (44)	81 (42.9)
Anti-topoisomerase I Ab, n (%)	421 (51)	48 (43)	55 (54)	222 (54)	93 (49.2)
Anti-centromere Ab, n (%)	141 (17)	19 (17)	20 (19)	60 (15)	40 (21.2)
Anti-RNA polymerase III Ab, n (%)	23 (3)	3 (3)	3 (3)	10 (3)	9.0 (8.6)
Total observation period, years (SD)	5.4 (2.0)	5.5 (2.1)	5.5 (2.1)	5.4 (2.0)	50 (26.5)
**Lung characteristics**					
FVC% predicted, mean (SD)	87 (21.1)	96 (23.1)	89 (21.4)	85 (20.6)	85.1 (19.5)
DL_{CO}% predicted, mean (SD)	59 (18.3)	62 (17.8)	58 (16.5)	58 (19.5)	59.0 (16.8)
∆FVC% predicted,* mean (SD)	–0.1 (10.2)	–17 (8.0)	–7 (1.4)	0.3 (2.5)	11.0 (7.3)
∆DL_{CO}% predicted,* mean (SD)	–0.7 (12.2)	–7 (17.2)	1 (10.0)	–0.4 (11.3)	2.4 (1.7)
Pulmonary hypertension, n (%)	162 (20)	21 (25)	16 (15)	6 (21)	30 (16)
NYHA class, n (%)					
1	363 (44)	53 (47)	46 (43)	175 (43)	89 (47)
2	317 (38)	44 (39)	41 (39)	154 (38)	77 (41)
3	103 (13)	12 (11)	14 (13)	52 (13)	24 (13)
4	14 (2)	3 (3)	1 (1)	8 (2)	2 (1)
**Other characteristics**					
mRSS, mean (SD)	10 (8.1)	11 (8.0)	9 (8.3)	10 (7.6)	9.9 (8.9)

Supplemental material BMJ Publishing Group Limited (BMJ) disclaims all liability and responsibility arising from any reliance placed on this supplemental material which has been supplied by the author(s) Ann Rheum Dis

	**Total (N=826)**	**Significant progression (n=113)**	**Moderate progression (n=106)**	**Stable (n=408)**	**Moderate improvement (n=189)**
∆mRSS, mean (SD)*	–0.4 (4.6)	0.5 (4.2)	–0.4 (3.2)	–0.4 (4.6)	–1.2 (5.3)
Reflux/dysphagia symptoms, n (%)	547 (66)	87 (77)	68 (64)	268 (66)	118 (62.4)
Digital ulcers, n (%)	266 (32)	43 (38)	28 (26)	144 (35)	60 (32)
Tendon friction rubs, n (%)	73 (9)	8 (7)	8 (8)	36 (9)	20 (11)
Synovitis, n (%)	117 (14)	18 (16)	13 (12)	62 (15)	23 (12)
Muscle weakness, n (%)	182 (22)	29 (26)	24 (23)	83 (20)	47 (25)
Scleroderma renal crisis, n (%)	11 (1)	4 (4)	3 (3)	6 (2)	1 (0.5)
ESR, mean (SD)	26 (20.6)	29 (23.8)	25 (21.9)	26 (19.0)	25.9 (20.8)
Elevated CRP, n (%)	217 (26)	38 (34)	32 (30)	102 (25)	47 (25)
Immunosuppressant use, n ($)	88 (11)	8 (7)	9 (9)	50 (12)	21 (11)

*Change from baseline to 12 months.

Significant progression (FVC decline of >10%, or FVC decline of 5–10% with DL_{CO} decline of ≥15%); moderate progression (FVC decline of 5–10% with DL_{CO} decline of <15%); stable ILD (FVC decline or improvement of <5%); moderate improvement (FVC improvement of 5–10% with DL_{CO} improvement of <15%).

Abbreviations: Ab, antibody; CRP, C-reactive protein; DL_{CO}, diffusion capacity of the lungs for carbon monoxide; ESR, erythrocyte sedimentation rate; FVC, forced vital capacity; mRSS, modified Rodnan skin score; NYHA, New York Heart Association; SD, standard deviation; SSc, systemic sclerosis; SSc-ILD, systemic sclerosis-associated interstitial lung disease.

Hoffmann-Vold A-M, *et al. Ann Rheum Dis* 2021; 80:219–227. doi: 10.1136/annrheumdis-2020-217455

BMJ Publishing Group Limited (BMJ) disclaims all liability and responsibility arising from any reliance placed on this supplemental material which has been supplied by the author(s)

SUPPLEMENTARY FIGURE S1 Variables predictive for progression in patients during the 12±3-month observation period in (A) univariable and (B) multivariable logistic regression; and variables predictive for further progression over the 5-year follow-up in patients with progression during the 12±3-month observation period in (C) univariable and (D) multivariable logistic regression.

Significant progression was defined as FVC decline of >10%, or FVC decline of 5–10% with DL_{CO} decline of ≥15%.

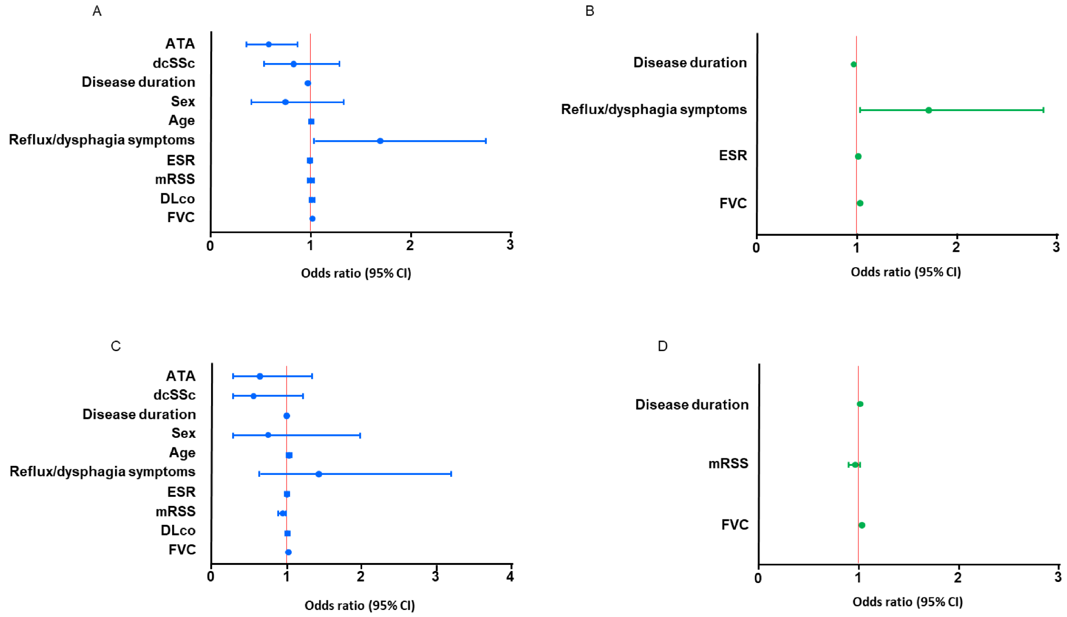

Abbreviations: ATA, anti-topoisomerase A antibody; CI, confidence interval; dcSSc, diffuse cutaneous systemic sclerosis; DL_{CO}, diffusion capacity of the lungs for carbon monoxide; ESR, erythrocyte sedimentation rate; FVC, forced vital capacity; mRSS, modified Rodnan skin score.

Hoffmann-Vold A-M, *et al. Ann Rheum Dis* 2021; 80:219–227. doi: 10.1136/annrheumdis-2020-217455

Appendix:

List of EUSTAR Collaborators

**Marco Matucci Cerinic**	Florence (Italy)
**Ulrich Walker**	Basel (Switzerland)
**Florenzo Iannone**	Bari (Italy)
**Radim Becvar**	Prague (Czech Republic)
**Gabriele Valentini**	Naples (Italy)
**Elise Siegert**	Berlin (Germany)
**C. Montecucco**	Pavia (Italy)
**Patricia E. Carreira**	Madrid (Spain)
**Carlo Chizzolini**	Geneva (Switzerland)
**Eugene J. Kucharz**	Katowice (Poland)
**Andrea Doria**	Padova (Italy)
**Pr Dominique Farge Bancel**	Paris (France)
**Roger Hesselstrand**	Lund (Sweden)
**Alexandra Balbir-Gurman**	Haifa (Israel)
**Raffaele Pellerito**	Torino (Italy)
**Cristian Caimmi**	Verona (Italy)
**Christopher Denton**	London (United Kingdom)
**Nemanja Damjanov**	Belgrade (Serbia & Montenegro)
**Jörg Henes**	Tübingen (Germany)
**Vera Ortiz-Santamaria**	Granollers Barcelona (Spain)
**Stefan Heitmann**	Stuttgart (Germany)
**Maria João Salvador**	Coimbra (Portugal)
**Bojana Stamenkovic**	Niska Banja (Serbia and Montenegro)
**Carlo Francesco Selmi**	Rozzano, Milano (Italy)
**Ariane Herrick**	Salford (United Kingdom)
**Ulf Müller-Ladner**	Bad Nauheim (Germany)
**Merete Engelhart**	Hellerup (Denmark)
**Valeria Riccieri**	Roma (Italy)
**Ruxandra Maria Ionescu**	Bucharest (Romania)
**Ana Maria Gheorghiu**	Bucharest (Romania)
**Cord Sunderkötter**	Münster (Germany)
**Jörg Distler**	Erlangen (Germany)
**Francesca Ingegnoli**	Milano (Italy)
**Luc Mouthon**	Paris (France)
**Vanessa Smith**	Gent (Belgium)
**Francesco Paolo Cantatore**	Foggia (Italy)
**Susanne Ullman**	Copenhagen (Denmark)
**Maria Rosa Pozzi**	Monza (Italy)
**Piotr Wiland**	Wroclaw (Poland)
**Marie Vanthuyne**	Brussels (Belgium)
**Brigitte Krummel-Lorenz**<br>**Petra Saar**	Frankfurt (Germany)

**Kristine Herrmann**	Dresden (Germany)
**Ellen De Langhe**	Leuven (Belgium)
**Branimir Anic**<br>**Marko Baresic**<br>**Miroslav Mayer**	Zagreb (Croatia)
**Sule Yavuz**	Altunizade-Istanbul (Turkey)
**Carolina de Souza Müller**	Curitiba (Brasil)
**Thierry Zenone**	Valence (France)
**Alessandro Mathieu**<br>**Alessandra Vacca**	Monserrato (CA) (Italy)
**Kamal Solanki**	Hamilton (New Zealand)
**Edoardo Rosato**	Roma (Italy)
**Fahrettin Oksel**<br>**Figen Yargucu**	Bornova, Izmir (Turkey)
**Cristina-Mihaela Tanaseanu**	Bucharest (Romania)
**Rosario Foti**	Catania (Italy)
**Daniel E. Furst**	Los Angeles (USA)
**Peter Villiger**<br>**Sabine Adler**	Bern (Switzerland)
**Paloma García de la Peña Lefebvre**<br>**Jorge Juan González Martín**	Madrid (Spain)
**Ira Litinsky**	Tel-Aviv (Israel)
**Francesco Del Galdo**	Leeds (United Kingdom)
**Goda Seskute**	Vilnius (Lithuania)
**Lesley Ann Saketkoo**	New Orleans (USA)
**Eduardo Kerzberg**	Buenos Aires (Argentina)
**Ivan Castellví**	Barcelona (Spain)
**François Spertini**	Lausanne (Switzerland)
**Vivien M. Hsu**	New Brunswick (USA)
**Thierry Martin**	Strasbourg (France)
**Tim Schmeiser**	Wuppertal-Elberfeld (Germany)
**Dominik Majewski**	Poznan (Poland)
**Vera Bernardino**	Lisboa (Portugal)
**Piercarlo Sarzi Puttini**	Milano (Italy)
